# Effectiveness of a Television Advertisement Campaign on Giving Cigarettes in a Chinese Population

**DOI:** 10.2188/jea.JE20130172

**Published:** 2014-11-05

**Authors:** Yu Qin, Jian Su, Quanyong Xiang, Yihe Hu, Guanqun Xu, Jiuhua Ma, Zumin Shi

**Affiliations:** 1 Jiangsu Provincial Center for Disease Control and Prevention, Nanjing, Jiangsu Province, P. R. China; 2 Suzhou Center for Disease Control and Prevention, Suzhou, Jiangsu Province, P. R. China; 3 Yizheng Center for Disease Control and Prevention, Yangzhou, Jiangsu Province, P. R. China; 4 Discipline of Medicine, University of Adelaide, Australia

**Keywords:** anti-tobacco television campaign, giving cigarettes, tobacco control

## Abstract

**Background:**

Anti-tobacco television advertisement campaigns may convey messages on smoking-related health consequences and create norms against giving cigarettes.

**Methods:**

Altogether, 156 and 112 slots of a television advertisement “Giving cigarettes is giving harm” were aired on Suzhou and Yizheng, respectively, over one month in 2010. Participants were recruited from 15 locations in Suzhou and 8 locations in Yizheng using a street intercept method. Overall 2306 residents aged 18–45 years completed questionnaires, including 1142 before the campaign and 1164 after, with respective response rates of 79.1% and 79.7%. Chi square tests were used to compare the difference between categorical variables.

**Results:**

After the campaign, 36.0% of subjects recalled that they had seen the advertisement. Residents of Suzhou had a higher recall rate than those of Yizheng (47.6% vs. 20.6%, *P* < 0.001). The rate of not giving cigarettes dropped from 32.1% before the campaign to 28.5% after (*P* = 0.05). In the post-campaign evaluation, participants who reported seeing the advertisement were more likely not to give cigarettes in the future than those who reported not seeing the advertisement (38.7% vs. 27.5%, *P* < 0.001).

**Conclusions:**

Our study showed that an anti-tobacco television advertisements helped change societal norms and improve health behavior. Continuous and adequate funding of anti-tobacco media campaigns targeted at different levels of the general population is needed, in conjunction with a comprehensive tobacco control effort.

## INTRODUCTION

Giving cartons (unopened multiple packs) of cigarettes as gifts is widespread in China, functioning as a good medium for upholding connections and maintaining mutually beneficial business associations in Chinese culture.^1^ Usually, cigarettes are given for special occasions and major holidays,^2^ such as wedding banquets and the Spring Festival (Chinese Lunar New Year). However, smoking-related health consequences are rarely considered when giving cigarettes.^1^ In 2011, only 23% of Chinese people knew that smoking can cause heart disease and stroke,^3^ which are two major causes of premature death in China.^4^ The truth regarding smoking consequences is usually ignored: there are 301 million smokers, 740 million passive smokers, and about 1 million smoking-related deaths every year in China.^3^^,^^5^

Mass media interventions often involve campaigns by television, radio, newspapers, billboards, posters, leaflets, or booklets. Television campaigns can convey messages on smoking-related health consequences and hardships through both graphic images and emotional content.^6^ Studies have shown that televised advertisements can increase intentions to quit, reduce smoking prevalence, generate calls to the quit line, and enhance tobacco control policy implementation.^7^^–^^9^

In 2008, the World Lung Foundation produced a televised advertisement “Giving cigarettes is giving harm”, which has been tested and shown to effectively change knowledge and behavior. Using this advertisement, the Jiangsu Provincial Center for Disease Control and Prevention launched a one-month television campaign to create norms against giving cigarettes during the Chinese Spring Festival in two cities of Jiangsu Province in 2010.

## METHODS

### Study design

The study was conducted in Suzhou and Yizheng of Jiangsu Province. Suzhou City lies in the southeastern area of Jiangsu Province, with a population of 5.7 million and a gross domestic product (GDP) of 900 billion Yuan Renminbi (RMB) in 2010. Yizheng County is part of Yangzhou City, located in the western area of Jiangsu Province, with a population of 593 000 and a GDP of 26.1 billion Yuan RMB in 2010.

The advertisement campaign “Giving cigarettes is giving harm” was aired on local television in each city for at least 30 days during January and February 2010. A street-intercept survey aimed at 18- to 45-year-old men and women was carried out in each city before and after the campaign.

### “Giving cigarettes is giving harm” television campaign

The advertisement, “Giving cigarettes is giving harm,” was provided by the Lung Foundation. It had two versions: one which was 30 seconds long and another 15 seconds. In the advertisement, people give cartons of cigarettes as gifts to their friends and family members. Simultaneously, a voice-over and caption delivers the following anti-smoking message:

“You have sent your friends both blessings and respiratory problems such as lung cancer. You have sent your colleagues both respect and cardiovascular and cerebrovascular disease. You have sent your family members both love and death. Giving cigarettes is giving harm.”

Seven programs on 4 television stations in Suzhou aired the 15-second advertisement for 32 consecutive days, with 165 slots from January 16 to February 27, 2010. The highest audience rating was 6.9%, and the lowest was 0.9% among the seven programs, according to data from the local cable television monitoring network. It was estimated that 165 742 out of 2 402 061 residents who were covered directly by Suzhou Television Station viewed the advertisement at least once. Two channels operated by Yizheng Television Station concurrently aired the advertisement between January 15 and February 12, 2010. It lasted for 28 days with 2 slots (one 15-second and one 30-second slot) per day on each channel, for a total of 112 slots altogether. No audience rating was provided.

In addition, newspapers, television stations and local website of the two cities reported the story. About 160 “Giving cigarettes is giving harm” posters with “You have sent your friends both blessings and respiratory problems such as lung cancer.” were displayed in public places such as communities, hospitals, schools, and bus or train stations in the two cities.

### Sampling plan

The survey and sampling protocol was provided by the World Lung Foundation. Five hundred subjects were invited to participate in the survey, approximately half smokers and half non-smokers. Participants were randomly selected using a street intercept method. In order to get a representative sample of the local residents, Suzhou and Yizheng were classified into five and four parts, respectively, by geographic area of each city. In each of these parts, two to three heavily trafficked spots were selected for collection of data. These areas included supermarkets, department stores, train stations, resident areas, bus terminals, hospitals, colleges/universities, market places, cultural centers, street intersections, parks, and restaurants. Altogether, 15 locations in Suzhou and 8 locations in Yizheng were selected for recruiting subjects. The study was approved by the ethical board of Jiangsu Provincial Center for Disease Control and Prevention. Verbal consent to take part was obtained from each participant.

### Subjects

Trained interviewers approached every fifth adult person who passed their way at the selected spots to participate in the survey. Of the subjects approached who appeared to be aged between 18–45 years, 2306 local residents completed questionnaires, including 1142 before and 1164 after the campaign. At the baseline survey, 46 subjects refused, 243 were beyond the age range, 11 from outside or living no more than 6 months in the two cities, and 1 with an incomplete questionnaire, resulting in a response rate of 79.1% (1142/1443). At the post-evaluation survey, 59 subjects refused, 199 were beyond the age range, 36 from outside or living no more than 6 months in the two cities, and 3 with incomplete questionnaires, resulting in a response rate of 79.7% (1164/1461). There was no difference in response rate between the two surveys (*P* = 0.75).

### Questionnaire

The questionnaire inquired about basic demographics such as age, gender, and education, as well as smoking status. Smoking status was defined as smoking cigarette in the past 30 days, and categorized into daily smoking, less than daily smoking, and never smoking (including ever smokers who had quit at least one month prior). Education level was divided into three groups: low (primary school, junior, and senior high school), medium (vocational or technical secondary school and junior college), and high (university and above).

Cigarette giving was assessed by two questions in all of the participants: ‘Have you ever given cigarettes as gifts to others?’, and, ‘How likely are you to give cigarettes as a gift in the future?’, the latter of which included possible responses of “not at all likely,” “somewhat likely,” “moderately likely,” “very likely,” and “extremely likely”. Information about adult tobacco use and attitude about and knowledge of diseases caused by smoking cigarettes were also evaluated.

During the post-evaluation, interviewees were shown a card with four shots of the advertisement and were then asked whether they had seen the advertisement and, if so, where they had seen it. Respondents were subsequently asked about attitudes regarding smoking.

### Statistical analysis

Chi square tests were used to compare the difference between categorical variables. All analyses were performed using IBM SPSS Statistics 19.0 (IBM Corporation, Armonk, NY, USA). Statistical significance was set at *P* < 0.05 (two sided).

## RESULTS

Overall, 2306 subjects completed the questionnaire: 1142 before the campaign and 1164 after. Table 1 shows the characteristics of the subjects before and after the campaign. There were no differences in living area, age, gender, rate of current smokers, and educational level between the two groups. The total proportion of those having ever given cigarettes to others was 51.1%, with no difference between subjects before (51.6%) or after (50.6%) the campaign.

**Table 1.** Characteristics of subjects before and after the campaign

		Before the campaign	After the campaign	*P* value
Total number of subjects		1142	1164	
Number of subjects by area	Suzhou	642	664	
	Yizheng	500	500	0.688
Age, years	18–25	213	225	
	26–35	402	404	
	36–45	527	535	0.912
Gender	Male	793	799	
	Female	349	365	0.679
Current smokers	No	564	545	
	Yes	578	619	0.218
Educational level^a^	Low	591	656	
	Medium	339	316	
	High	212	192	0.083
Has ever given cigarettes	No	553	575	
	Yes	589	589	0.640

^a^Low: primary school or junior and senior high school; medium: vocational or technical secondary school or junior college; high: university or above.

After the campaign, 36.0% of subjects recalled that they had seen the advertisement. There was a significant regional difference in the recall rate: 47.6% and 20.6% in Suzhou and Yizheng, respectively (*P* < 0.001). Males had a higher recall rate than females (39.0% vs. 29.3%, *P* = 0.001). The recall rate increased with education level (*P* = 0.005). However, the recall rate did not vary by current smoking status or history of giving cigarettes (Table 2).

**Table 2.** Recall of the advertisement “giving cigarette is giving harm”

		Recall rate of the advertisement (%)	*P* value
Overall		36.0	
Area	Suzhou	47.6	
	Yizheng	20.6	<0.001
Age, years	18–25	36.9	
	26–35	36.9	
	36–45	35.0	0.544
Gender	Male	39.0	
	Female	29.3	0.001
Current smoker	No	33.8	
	Yes	38.0	0.136
Educational level^a^	Low	31.7	
	Medium	42.7	
	High	39.6	0.005
Has ever given cigarettes	No	33.4	
	Yes	38.6	0.067

^a^Low: primary school or junior and senior high school; medium: vocational or technical secondary school or junior college; high: university or above.

Of those who saw the advertisement, 68.7% reported that the advertisement made them stop and think; 74.7% reported that it was relevant to them and their life; 73.3% thought that it provided new information. After watching the advertisement, 57.8% of subjects desired to say something about it or discuss it with others; 48.4% decided not to give cigarette as gifts to anyone in the future; 40.6% tried to persuade others to not give cigarettes as gifts; and 45.3% tried to persuade others to quit smoking. Among smokers who had seen the advertisement, 51.9% made an attempt to quit smoking. After the campaign, participants were more likely not to give cigarettes as gifts in the future: the rate dropped from 32.1% (before campaign) to 28.5% (*P* = 0.05). Subjects with no history of giving cigarettes had the highest rate of not giving cigarettes in the future before and after the campaign (54.8% and 60.2%, respectively). However, participants with a history of giving cigarettes had the lowest proportion of not intending to give cigarettes in the future (less than 5% both before and after the campaign). Subjects with a high education level had the highest change in proportion of those who were not intending to give cigarettes in the future after the campaign (42.1%), while young subjects aged 18–25 years and non-smokers had the lowest change (1.8%; Table 3). In the post-campaign evaluation, participants who reported seeing the advertisement were more likely not to intend to give cigarettes in the future than those who reported not seeing the advertisement (38.7% vs. 27.5%, *P* < 0.001).

**Table 3.** Proportion intending to not give cigarettes in the future before and after the campaign

		Before the campaign	After the campaign	Relative change (%)
Overall		28.5	32.1	12.6
Area	Suzhou	36.0	39.3	11.6
	Yizheng	18.8	22.6	13.3
Age, years	18–25	31.9	32.4	1.8
	26–35	27.1	28.5	4.9
	36–45	28.1	34.8	23.5
Gender	Male	27.7	31.5	13.3
	Female	30.1	33.4	11.6
Current smokers	No	36.7	37.2	1.8
	Yes	20.4	27.6	25.3
Educational level^a^	Low	21.3	23.9	9.1
	Medium	33.9	37.3	11.9
	High	39.6	51.6	42.1
Has ever given cigarettes	No	54.8	60.2	18.9
	Yes	3.7	4.8	3.9

^a^Low: primary school or junior and senior high school; medium: vocational or technical secondary school or junior college; high: university or above.

Table 4 shows information regarding participants’ attitude regarding smoking and knowledge related to cigarettes before and after the campaign. More subjects after the campaign agreed that cigarettes are harmful gifts and that smoking cigarettes causes serious harm to one’s health than before the campaign. The proportion of subjects who disagreed that tobacco products make good gifts for people and that the dangers of smoking have been exaggerated did not differ before and after the campaign. More subjects after the campaign knew that smoking cigarettes cause lung disease, heart disease, stroke, and impotence than before the campaign, except regarding knowledge of the association between smoking cigarettes and oral cancer.

**Table 4.** Attitude and knowledge related to cigarette before and after the campaign

	Before the campaign (%)	After the campaign (%)	χ^2^	*P* value
Disagree that tobacco products make good gifts for people	53.9	55.2	0.393	0.531
Agree that cigarettes are harmful gifts	66.9	75.3	20.026	<0.001
Agree that smoking cigarettes causes serious harm to one’s health	82.3	85.4	4.051	0.044
Disagree that the dangers of smoking have been exaggerated	61.0	59.9	0.321	0.571
Know that smoking cigarettes cause:				
*Lung disease*	92.1	94.6	5.671	0.017
*Oral cancer*	48.2	50.2	0.853	0.356
*Heart disease*	37.0	45.6	17.489	<0.001
*Stroke*	22.0	32.5	32.01	<0.001
*Impotence*	16.1	26.5	37.34	<0.001

## DISCUSSION

As the first anti-tobacco television campaign and evaluation in Jiangsu Province, our study showed that a one-month television advertising campaign (with a recall rate of 36.0%) decreased the intention to give cigarettes as gifts and improved knowledge on tobacco hazards. However, two out of three respondents stated that they would still give cigarettes as gifts, and nearly half of participants regarded cigarettes as good gifts. The knowledge of smoke-related cardiovascular disease and stroke was quite low.

More than half of adult males smoke in China, and the prevalence increases with age.^10^ Male smokers are accustomed to sharing cigarettes, and smoking in public places is usually acceptable. The prevalence of passive smoking is high and roughly equal among males and females,^3^ and knowledge of smoking-related health hazards is low in both smokers and non-smokers, even those with a high education level.^11^ Although the prevalence of awareness of smoking-related diseases increased due to the campaign in our study, it remains low. This result is consistent with national reports.^11^

To effectively control smoking, China officially ratified the WHO Framework Convention on Tobacco Control (FCTC), which took effect on January 9, 2006. Six key measures to assist in country-level implementation of WHO FCTC are described in WHO’s 2008 MPOWER package to counter the tobacco epidemic and reduce its deadly toll.^12^ Using the MPOWER package, the score was only 37.3 on a 100-point scale in China (evaluated in 2011).^13^ The score signifies considerable failure to meet FCTC requirements, which demand not only packaging and labelling measures, but also a comprehensive ban on all direct and indirect tobacco advertising, promotion, and sponsorship in both the traditional media and all other media platforms.^5^

Smoking serves an important social function in reinforcing friendships and relationships in China.^14^ Giving cigarettes is rooted in Chinese culture. A survey showed that respondents thought cigarettes were good gifts partly because they are portable, easy to purchase, and nonperishable.^2^ National and international tobacco companies design attractive packaging, which serves as both advertising and making cigarettes suitable as gifts.^15^ A previous study in two other cities of Jiangsu Province showed that people would not give cigarettes as gifts if the warnings on cigarette package were large, clear, and legible with a picture showing diseases and suffering caused by tobacco use.^16^ Despite FCTC calls for a comprehensive ban on tobacco advertising, promotion, and sponsorship, implementation remains a challenge.^17^^,^^18^ About 20% of Chinese people have ever seen direct advertising, with approximately half of these advertisements seen on TV programs.^10^ Prevalent tobacco advertisement in retail outlets and a high density of tobacco retail outlets was found in Hangzhou.^19^ In addition, tobacco companies frequently present advertising in mass media, sports events, film, and TV programs, increasing their influence through donations and sponsorship.^20^^,^^21^ These efforts reinforce the social norm of giving cigarettes, maintain the social acceptability of smoking, and further encourage more smoking while decreasing the motivation of smokers to quit.^15^

An effective media campaign is a key element of tobacco control. An Australian study showed that television advertisements increased awareness of the health consequences of smoking and motivation to quit.^22^ Increased cessation and reduced smoking rates was reported when The New York Tobacco Control Program Campaigns were launched from 2003–2009, which increased smokers’ exposure to anti-tobacco advertising from 6% to 45%.^7^ Campaigns of longer duration and higher intensity appear to be more effective than shorter or less intense campaigns, with greater declines in smoking rates and increases in smoking cessation.^8^^,^^23^^,^^24^ The mass media campaigns may be more effective when accompanied by other policies, such as smoking bans in public places and workplaces.^25^^,^^26^ Our study only showed a positive effect on the norm of giving cigarettes using a short-term television advertisement. The long-term effects of the mass media campaign used in the present study remain unknown.

In our study, Yizheng (a less developed area) had a lower recall rate of the advertisement and higher cigarette giving than Suzhou (a more developed area). The discrepancy may be due to more slots with more TV stations aired in Suzhou than Yizheng. In fact, a high prevalence of cigarette smoking, low education levels, and less access to health messages coexist in rural areas in China.^3^ Although younger people (<35 years old) had a high recall rate in our study, they were less likely to change their behavior of giving cigarettes than older people (>35 years old). Notably, the younger generation is more likely to start smoking because of tobacco advertisement, promotion, and sponsorship.^27^^,^^28^

There are some limitations in this study. First, because of our sampling method, caution should be taken when generalizing these results to the whole population. However, as an impact evaluation study targeting communities, we systematically selected 8–12 spots at different geographic points of the two cities to capture different demographic groups, which aimed to create a representative sample. This street intercept methodology has been used for other public health studies, particularly in low-resource countries or to recruit harder-to-reach populations.^29^^–^^32^ Second, the subjects involved in the study were aged 18–45 years, and the results may not be generalizable to older people. However, younger people are at a higher risk of giving cigarettes, as older people usually receive cigarettes as gifts rather than give them.^33^ Third, the influence of our media campaign may depend on other interventions already in place before the campaign began. Existing campaigns, such as smoke-free areas in organizations providing health services, and community-based health education, may have already helped raise awareness of health consequences caused by cigarette smoking.

In conclusion, our study suggests that anti-tobacco television advertisements may help change norms and improve health behavior. Media campaigns may create new norms against giving cigarettes and increase awareness of cigarette harms and strategies for quitting. Continuous and adequate funding of anti-tobacco media campaigns targeted at different levels of the general population are needed, in conjunction with a comprehensive tobacco control effort. Strict enforcement of polices, such as pictorial warnings on tobacco packaging and bans on tobacco advertisement according to WHO FCTC guidelines, should also be strengthened.

## References

[1] Rich ZC, Xiao S. Tobacco as a social currency: cigarette gifting and sharing in China. Nicotine Tob Res. 2012;14:258–63. 10.1093/ntr/ntr156 21849412

[2] Hu M, Rich ZC, Luo D, Xiao S. Cigarette sharing and gifting in rural China: a focus group study. Nicotine Tob Res. 2012;14:361–7. 10.1093/ntr/ntr262 22218401

[3] Chinese Center for Disease Control and Prevention. Global adult tobacco survey (GATS) China 2010 country report. 2011.

[4] Yang G, Kong L, Zhao W, Wan X, Zhai Y, Chen LC, . Emergence of chronic non-communicable diseases in China. Lancet. 2008;372:1697–705. 10.1016/S0140-6736(08)61366-5 18930526

[5] Huang J, editor. Tobacco hazard and tobacco control. Beijing: Xinhua Publishing House; 2012.

[6] Wakefield M, Bayly M, Durkin S, Cotter T, Mullin S, Warne C; International Anti-Tobacco Advertisement Rating Study Team. Smokers’ responses to television advertisements about the serious harms of tobacco use: pre-testing results from 10 low- to middle-income countries. Tob Control. 2013;22:24–31. 10.1136/tobaccocontrol-2011-050171 21994276

[7] Davis KC, Farrelly MC, Duke J, Kelly L, Willett J. Antismoking media campaign and smoking cessation outcomes, New York State, 2003–2009. Prev Chronic Dis. 2012;9:E40. 22261250 PMC3320091

[8] Friend K, Levy DT. Reductions in smoking prevalence and cigarette consumption associated with mass-media campaigns. Health Educ Res. 2002;17:85–98. 10.1093/her/17.1.85 11888047

[9] Wilson N, Grigg M, Graham L, Cameron G. The effectiveness of television advertising campaigns on generating calls to a national Quitline by Maori. Tob Control. 2005;14:284–6. 10.1136/tc.2004.010009 16046693 PMC1748067

[10] Yang GH, Li Q, Wang CX, Hsia J, Yang Y, Xiao L, . Findings from 2010 Global Adult Tobacco Survey: implementation of MPOWER policy in China. Biomed Environ Sci. 2010;23:422–9. 10.1016/S0895-3988(11)60002-0 21315239

[11] Yang Y, Wang JJ, Wang CX, Li Q, Yang GH. Awareness of tobacco-related health hazards among adults in China. Biomed Environ Sci. 2010;23:437–44. 10.1016/S0895-3988(11)60004-4 21315241

[12] World Health Organization. WHO report on the global tobacco epidemic, 2008. The MPOWER package. Geneva: World Health Organization; 2008.

[13] Yang GH, Hu AG. Tobacco control and the future of China. The joint assessment of the tobacco use and tobacco control in China by internal and international experts. Beijing: Economic Daily Press; 2011.

[14] Pan Z. Socioeconomic predictors of smoking and smoking frequency in urban China: evidence of smoking as a social function. Health Promot Int. 2004;19:309–15. 10.1093/heapro/dah304 15306615

[15] Chu A, Jiang N, Glantz SA. Transnational tobacco industry promotion of the cigarette gifting custom in China. Tob Control. 2011;20:e3. 10.1136/tc.2010.038349 21282136 PMC3124594

[16] Qin Y, Wu M, Pan X, Xiang Q, Huang J, Gu Z, . Reactions of Chinese adults to warning labels on cigarette packages: a survey in Jiangsu Province. BMC Public Health. 2011;11:133. 10.1186/1471-2458-11-133 21349205 PMC3053246

[17] Centers for Disease Control and Prevention (CDC). Adult awareness of tobacco advertising, promotion, and sponsorship—14 countries. MMWR Morb Mortal Wkly Rep. 2012;61:365–9. 22622091

[18] Nagler RH, Viswanath K. Implementation and research priorities for FCTC Articles 13 and 16: tobacco advertising, promotion, and sponsorship and sales to and by minors. Nicotine Tob Res. 2013;15:832–46. 10.1093/ntr/nts331 23291641 PMC3601914

[19] Gong T, Lv J, Liu Q, Ren Y, Li L, Kawachi I. Audit of tobacco retail outlets in Hangzhou, China. Tob Control. 2011. 22180327 10.1136/tobaccocontrol-2011-050038

[20] Charlesworth A, Glantz SA. Smoking in the movies increases adolescent smoking: a review. Pediatrics. 2005;116:1516–28. 10.1542/peds.2005-0141 16322180

[21] Lv J, Su M, Hong Z, Zhang T, Huang X, Wang B, . Implementation of the WHO Framework Convention on Tobacco Control in mainland China. Tob Control. 2011;20:309–14. 10.1136/tc.2010.040352 21493635

[22] Brennan E, Durkin SJ, Cotter T, Harper T, Wakefield MA. Mass media campaigns designed to support new pictorial health warnings on cigarette packets: evidence of a complementary relationship. Tob Control. 2011;20:412–8. 10.1136/tc.2010.039321 21474501

[23] Bala M, Strzeszynski L, Cahill K. Mass media interventions for smoking cessation in adults. Cochrane Database Syst Rev. 2008;CD004704. 18254058 10.1002/14651858.CD004704.pub2

[24] Dietz NA, Westphal L, Arheart KL, Lee DJ, Huang Y, Sly DF, . Changes in youth cigarette use following the dismantling of an antitobacco media campaign in Florida. Prev Chronic Dis. 2010;7:A65. 20394704 PMC2879997

[25] Gagné L. The 2005 British Columbia Smoking Cessation Mass Media Campaign and short-term changes in smoking. J Public Health Manag Pract. 2007;13:296–306. 10.1097/01.PHH.0000267688.54024.09 17435497

[26] Goldman LK, Glantz SA. Evaluation of antismoking advertising campaigns. JAMA. 1998;279:772–7. 10.1001/jama.279.10.772 9508154

[27] Doku D, Raisamo S, Wiium N. The role of tobacco promoting and restraining factors in smoking intentions among Ghanaian youth. BMC Public Health. 2012;12:662. 10.1186/1471-2458-12-662 22894679 PMC3490846

[28] Hanewinkel R, Isensee B, Sargent JD, Morgenstern M. Cigarette advertising and adolescent smoking. Am J Prev Med. 2010;38:359–66. 10.1016/j.amepre.2009.12.036 20307803

[29] Gase LN, Robles B, Barragan NC, Kuo T. Relationship Between Nutritional Knowledge and the Amount of Sugar-Sweetened Beverages Consumed in Los Angeles County. Health Educ Behav. 2014. 10.1177/1090198114529128 24717193

[30] Jurawan K, Dindial D, Hosein S, King D, Sahadeo A, Mungrue K. An evaluation of intervention strategies for HIV/AIDS in Trinidad and Tobago (2000–2007). Int J Adolesc Med Health. 2009;21:581–9. 10.1515/IJAMH.2009.21.4.581 20306770

[31] Miller KW, Wilder LB, Stillman FA, Becker DM. The feasibility of a street-intercept survey method in an African-American community. Am J Public Health. 1997;87:655–8. 10.2105/AJPH.87.4.655 9146448 PMC1380849

[32] Poomsrikaew O, Ryan CJ, Zerwic JJ. Knowledge of heart attack symptoms and risk factors among native Thais: a street-intercept survey method. Int J Nurs Pract. 2010;16:492–8. 10.1111/j.1440-172X.2010.01874.x 20854347

[33] Huang LL, Thrasher JF, Jiang Y, Li Q, Fong GT, Quah AC. Incidence and correlates of receiving cigarettes as gifts and selecting preferred brand because it was gifted: findings from the ITC China Survey. BMC Public Health. 2012;12:996. 10.1186/1471-2458-12-996 23157697 PMC3532818

